# iRGD-TRP-PK1-modified red blood cell membrane vesicles as a new chemotherapeutic drug delivery and targeting system in head and neck cancer

**DOI:** 10.7150/thno.99481

**Published:** 2025-01-01

**Authors:** Suwen Bai, Zunyun Wang, Yuxin Zhang, Yunyun Yang, Yuan Wei, Yumei Luo, Minghua Wang, Bing Shen, Wei He, Zhenye Yang, Hui Hui, Juan Du

**Affiliations:** 1 The Second Affiliated Hospital, School of Medicine, The Chinese University of Hong Kong, Shenzhen & Longgang District People's Hospital of Shenzhen, Shenzhen, 518172, China.; 2 Ciechanover Institute of Precision and Regenerative Medicine, School of Medicine, The Chinese University of Hong Kong, Shenzhen, Guangdong, 518172, China.; 3 School of Basic Medical Sciences, Anhui Medical University, Hefei, Anhui, 230032, China.; 4 CAS Key Laboratory of Molecular Imaging, Institute of Automation, Beijing, 100190, China.; 5 Dr. Neher's Biophysics Laboratory for Innovative Drug Discovery State Key Laboratory of Quality Research in Chinese Medicine.; 6 Key Laboratory of Immune Response and Immunotherapy, School of Basic Medical Sciences, Division of Life Sciences and Medicine, University of Science and Technology of China, Hefei, China.; 7 National Key Laboratory of Kidney Diseases, Beijing, 100853, China

**Keywords:** Drug Delivery, Head and Neck Cancer, iRGD-TRP-PK1, Red Blood Cell Membrane Vesicles, Targeting System

## Abstract

**Background:** Chemotherapy is essential for treating tumors, including head and neck cancer (HNC). However, the toxic side effects of chemotherapeutic drugs limit their widespread use. Therefore, a targeted delivery system that can transport the drug to the pathological site while minimizing damage to healthy tissues is urgently needed.

**Methods:** Application of animal imaging, flow cytometry, fluorescence staining, cell activity assay, transmission electron microscopy, western blotting and immunohistochemistry to evaluate the targeting and killing effects of internalizing RGD peptide (iRGD)-transient receptor potential (TRP)-PK1-modified red blood cell vesicles (RBCVs) on HNC cells *in vitro* and *in vivo*.

**Results:** TRP-PK1 was ligated to iRGD, enabling autonomous insertion into the lipid bilayer. Additionally, RBCVs were labeled with iRGD-TRP-PK1 to achieve tumor targeting. Based on the self-assembly capability of TRP-PK1 to form a “leakage potassium” channel on the biofilm, RBCVs were fragmented within the high-potassium (K^+^) environment inside tumor cells. This fragmentation facilitated the release of the drug loaded onto the RBCVs.

**Conclusion:** The advantageous properties of TRP-PK1 are utilized in our design, resulting in a cost-effective and straightforward approach to drug delivery and release. Ultimately, the objective of suppressing tumor growth while minimizing side effects was accomplished by iRGD-TRP-PK1-modified RBCVs in our study. These findings provide novel insights into the enhancement of targeted delivery systems and present promising avenues for the treatment of HNC.

## Introduction

Head and neck cancer (HNC) is a malignant tumor located in the upper aerodigestive tract, with approximately 890,000 new cases reported worldwide annually 1. HNC is generally discovered in the later stages of tumor progression 2. While surgery is the main treatment for HNC, many patients experience locally advanced disease and require additional chemotherapy 3. Most chemotherapy drugs cause side effects such as hair loss, bone marrow suppression, liver and kidney dysfunction, and cardiotoxicity 4. Therefore, it is urgent to adopt therapeutic protocols that can reduce toxicity and maintain quality of life without significantly compromising efficacy. To address the toxic side effects of chemotherapy drugs, various nanoparticle carrier models have been engineered with tumor tissue-targeted ligand modifications. Those particles can transport chemotherapy drugs directly into tumors 5. Among these nanoparticle carrier models, the tumor-penetrating internalized RGD peptide (iRGD) has attracted significant attention due to its superior tumor targeting and penetration capabilities 6,7.

iRGD (CRGDKGPDC) is a cyclic peptide containing a protease recognition site, a CendR tissue penetration motif (CRGDR/K), and a tumor/neovascular-specific RGD motif 5,6,8-10. Consequently, iRGD-modified nanoparticles can specifically recognize and bind to αvβ integrins, which are highly expressed on tumor cells and tumor neovascular endothelial cells. This property facilitates the targeted delivery of iRGD-modified nanoparticles to tumor tissues 11. One of the primary strategies to enhance the therapeutic efficacy of chemotherapy through iRGD technology involves constructing drug carriers with tumor-targeting properties by combining iRGDs with vectors. Subsequently, chemotherapeutic drugs are loaded onto these carriers to augment their therapeutic effectiveness 12. Although iRGD-modification can improve the tumor targeting of nanodelivery systems, the majority of these modifications are achieved through chemical coupling and genetic engineering technology. However, this method typically involves a complex preparation process, and the conjugation efficiency and yield may be limited 6,10,13,14. Furthermore, this complex preparation method is expensive, time-consuming, environmentally unfriendly, and may not be conducive to clinical translation. Additionally, it could potentially affect the activity of iRGD 11. Therefore, it is essential to develop new methods to address these challenges.

We have identified a synthetic peptide derived from the fourth transmembrane fragment of transient receptor potential vanilloid 4 (TRPV4), named TRP-PK1, which possesses the ability to autonomously insert itself into the cell membrane and form a potassium (K^+^)-like ion channel 15. Based on this property, this study aimed to link iRGD with TRP-PK1, resulting in iRGD-TRP-PK1. Subsequently, vesicles prepared from red blood cell membranes (RBCVs) were utilized as carriers for chemotherapeutic drugs. The RBCVs were then co-incubated with iRGD-TRP-PK1, enabling the autonomous labeling of RBCVs with the targeted peptide, iRGD. This process aimed to facilitate the targeting and transportation of RBCVs into tumor tissues to exert anti-tumor effects.

## Materials and Methods

### Cell culture

Normal human nasopharyngeal epithelial cell line-NP69 and the nasopharyngeal carcinoma cell lines CNE2, 5-8F, HONE1, and CNE1 were cultured in Roswell Park Memorial Institute 1640 cell culture medium supplemented with 10 % fetal bovine serum (FBS) and 1% antibiotics (100 kU/L penicillin and 100 mg/L streptomycin) (VivaCell, Shanghai, China) 16. HN4 cells, derived from a patient with squamous cell carcinoma of the head and neck, were cultured in Dulbecco's modified Eagle's medium (DMEM) (glucose concentration is 4.5 g/L) supplemented with 10% FBS and 1% antibiotics (100 kU/L penicillin and 100 mg/L streptomycin) 17. All cells were maintained at 37 ℃ in a humidity-controlled incubator with 5% CO_2_.

### iRGD-TRP-PK1-modified red blood cell membrane vesicle production

***RBC membrane isolation and RBCV preparation:*** Approximately 1 mL of BALB/c mice blood was collected in an anticoagulation tube. The blood was then centrifuged at 2,000 r.p.m. for 10 min at 4 ℃, and the blood plasma was discarded. The blood cells were subsequently washed thrice with phosphate-buffered saline (PBS). Erythrocyte membrane fragments were obtained from the RBCs by subjecting them to a 40 mL hypotonic (10 mM Tris-HCl, pH=7.4) treatment for 2 h at 4 ℃. The resulting mixture was centrifuged at 3,500 r.p.m. for 5 min at 4 ℃ to remove hemoglobin. The erythrocyte membrane were then collected and washed thrice with PBS. To obtain intact RBCVs, erythrocyte membranes were placed in isotonic solution (PBS) overnight at 4 ℃.

***Preparation of iRGD-TRP-PK1-modified RBCV:*** RBCs were co-incubated with iRGD-TRP-PK1 (Synthesized by QYAOBIO, Shanghai, China) at 4 ℃ overnight. Subsequently, erythrocyte membranes were extruded through 400 nm, 200 nm, and 100 nm polycarbonate porous membranes using Avanti mini extruder (AvantiPolar Lipids) to obtain RBCVs.

### Characterization of RBCV

After isolating RBCVs and preparing iRGD-TRP-PK1-modified RBCVs, the size, zeta potential and concentration of the samples were measured using Nanocoulter (Resuntech, Shenzhen, China). Additionally, sample solutions were fixed on copper grids, stained for 2 min, and air-dried. Finally, transmission electron microscopy (FEI, USA) was utilized for morphological observations.

### Loading chemotherapy drug

To prepare chemotherapy drug-loaded RBCVs, we mixed 1 µM doxorubicin (Dox) or 3 µM cisplatin (CDDP) with RBCVs, and then incubated them at 4 ℃ overnight. Subsequently, PBS was used to remove free Dox or CDDP. The concentration of Dox or CDDP encapsulated in RBCVs was measured using an automated enzyme immunoassay analyzer (Agilent, USA) based on a calibration curve with fluorescence intensity at 535/590 nm excitation/emission wavelengths 18. The loading efficiency was calculated using the initial chemotherapeutic drug concentration (C_I_) and the final supernatant chemotherapeutic drug concentration (C_F_): loading efficiency=(C_I_ - C_F_)/C_I_ × 100%.

### Western blotting

The HNC cell proteins were extracted using radio-immunoprecipitation assay lysis buffer (Beyotime Biotechnology, Shanghai, China), and protein concentrations were determined using the Bicinchoninic acid kit. The subsequent steps followed our previously reported protocol 16. Primary antibodies used included anti-α_v_β_3_ (1:250, Bioss, Beijing, China), and anti-GAPDH (1:1000, Affinity Biosciences). Immunoreactive bands were detected using enhanced chemiluminescence (Tanon, Shanghai, China), and a chemiluminescence imaging system (Invitrogen iBright CL1000, USA) was used for image acquisition.

### TUNEL assay

Cell apoptosis was measured by TUNEL assay (Beyotime, Shanghai, China). Briefly, HNC cells were fixed with 4 % paraformaldehyde solution after appropriate experimental treatment, and then proteinase K solution (2 mg/ml) was used to inactivate endogenous peroxidase. Next, equilibration buffer was treated to cells for 20 min at room temperature. Cells were washed three times with PBS, and incubated with TdT buffer for 60 min at 37 ℃. HNC cells were stained with 4',6-diamidino-2-phenylindole (DAPI) for 10 min at room temperature after PBS washed three times. Finally, cells were dried and analyzed by fluorescence microscopy. Blue fluorescence at a wavelength of 460 nm (DAPI) visualizes each cell and green fluorescence at a wavelength of 520 nm identifies apoptotic cells. Percentage of apoptosis = number of apoptotic cells/total number of cells × 100 %.

### Flow cytometry

***Positive cell count after RBCV uptake:*** RBCV, iRGD-RBCV, TRP-PK1-RBCV, iRGD-TRP-PK1-RBCV, Cyclo(-RGDfK)+iRGD-TRP-PK1-RBCV (RBCVs were using PKH26 labeling) were treated HN4 cells for 24 h. Then digested cells are made into suspensions and tested directly on the FACS (BD, FACSCanto II).

***Cell apoptosis:*** The cells were harvested by trypsinization and washed three times with cold PBS after treated with free CDDP, RBCV+CDDP, iRGD-RBCV+CDDP, iRGD-TRP-PK1-RBCV+CDDP or Cyclo(-RGDfK)+iRGD-TRP-PK1-RBCV+CDDP. All group were referenced to the concentration of CDDP, 3 µM. Then the cells were centrifuged at 3000 r/min for 5 min at 4 ℃. Then the cells were resuspended in 195 µl Annexin V-FITC binding buffer, and added 10 µl PI and 5 µl Annexin V-FITC for 20 min at room temperature in the dark. Finally, added 300 µl PBS and analyzed by FACS (BD, FACSCanto II).

### Transmission electron microscopy (TEM)

RBCVs were fixed with glutaraldehyde (2.5%) at 4℃ overnight, followed by washing with PBS. Subsequently, the samples were stained with uranyl acetate (2%) for 10 min and then with lead stain for 5 min. Sections were examined under a TEM (FEI, USA) at a voltage of 120 kV. TEM images were captured using an Ouemesa CCD digital camera and processed using Olympus Soft Imaging software (Zeiss, German).

### Cell Counting Kit-8 (CCK-8) assay

The cells were seeded into 96-well culture plates at a density of 6×10^3^ cells/well. Subsequently, they were treated with RBCVs, iRGD-TRP-PK1-modified RBCVs, or RBCVs loaded with chemotherapy drugs. After 24 h, 100 μL of the medium was supplemented with 10 μL of CCK-8 (MedChemExpress, USA) and incubated for 4 h at 37 °C with 5% CO_2_. The absorbance was measured at 450 nm, and the amount of anthranilic acid dye produced was positively correlated with the number of surviving cells.

### Cellular uptake study

HN4 or CNE2 cells (1×10^4^) were cultured in a 12-well plate overnight. Subsequently, these cells were incubated with Phalloidin (Green) (Servicebio, Wuhan, China) and 4ʹ,6-diamidino-2-phenylindole (Blue) (Beyotime Biotechnology, Shanghai, China) to label the cell membrane and nucleus as instructed. Meanwhile, the cells were co-cultured with RBCVs alone, a mixture of RBCVs and TRP-PK1, and a mixture of RBCVs and iRGD-TRP-PK1, with RBCVs marked with Paul Karl Horan 26^th^ variant (PKH26) (Red) (MedChemExpress, USA). The cells were then observed using a fluorescence microscope (Zeiss, German).

### Release of Dox from RBCV in HNC cells

For *in vivo* release detection, iRGD-TRP-PK1-RBCV+Dox was co-cultured with HN4 or CNE2 cells for 0 h, 3 h, and 6 h. RBCVs were labeled with PKH67 (Green) (MedChemExpress, USA), and Dox exhibits spontaneous red fluorescence. The intracellular co-localization of red and green fluorescence was detected using a fluorescence microscope (Zeiss, German).

### Xenograft mouse model and treatment

All animal experiments were conducted per the guidelines of the Experimental Animal Center of Longgang District People's Hospital of Shenzhen and were approved by the Ethics Committee of Longgang District People's Hospital of Shenzhen. BALB/c nude mice (approximately 7 weeks old) were used to study the *in vivo* antitumor efficacy of iRGD-TRP-PK1-modified RBCVs loaded with Dox and CDDP. HN4 (1×10^6^) cells suspended in 50 µL of DMEM HG medium and mixed with matrix gel (1:1) (MedChemExpress, USA) were injected subcutaneously into mice. The mice were randomly divided into ten groups. Subsequently, they were intravenously injected with the following treatments once every three days: PBS (control group), iRGD-TRP-PK1 (2 mg/kg), RBCV (1 mg/kg), iRGD-TRP-PK1-RBCV (1 mg/kg, based on RBCV), iRGD-TRP-PK1-RBCV+Dox (1 mg/kg, based on Dox), iRGD-TRP-PK1-RBCV+CDDP (3 mg/kg, based on CDDP), Dox (1 mg/kg), CDDP (3 mg/kg), RBCV+Dox (1 mg/kg, based on Dox), and RBCV+CDDP (3 mg/kg, based on CDDP). Tumor volume was calculated using formula the formula: Tumor volumes (mm^3^) = 1/2 × length × width^2^. To assess tumor targeting, RBCVs were labeled with DiR (MedChemExpress, USA), and fluorescence intensity in the tumor tissue was detected using *In Vivo* Imagining System Spectrum (NIROPTICS, Suzhou, China).

### Statistical analysis

For statistical analysis, GraphPad Prism software 6.0 and Student's t-test were used. All data are presented as means ± standard error of the mean.

## Results

### Construction and characterization of iRGD-TRP-PK1-modified red blood cell vesicle (RBCV)-loaded anti-cancer drug system

**Figure 1:**
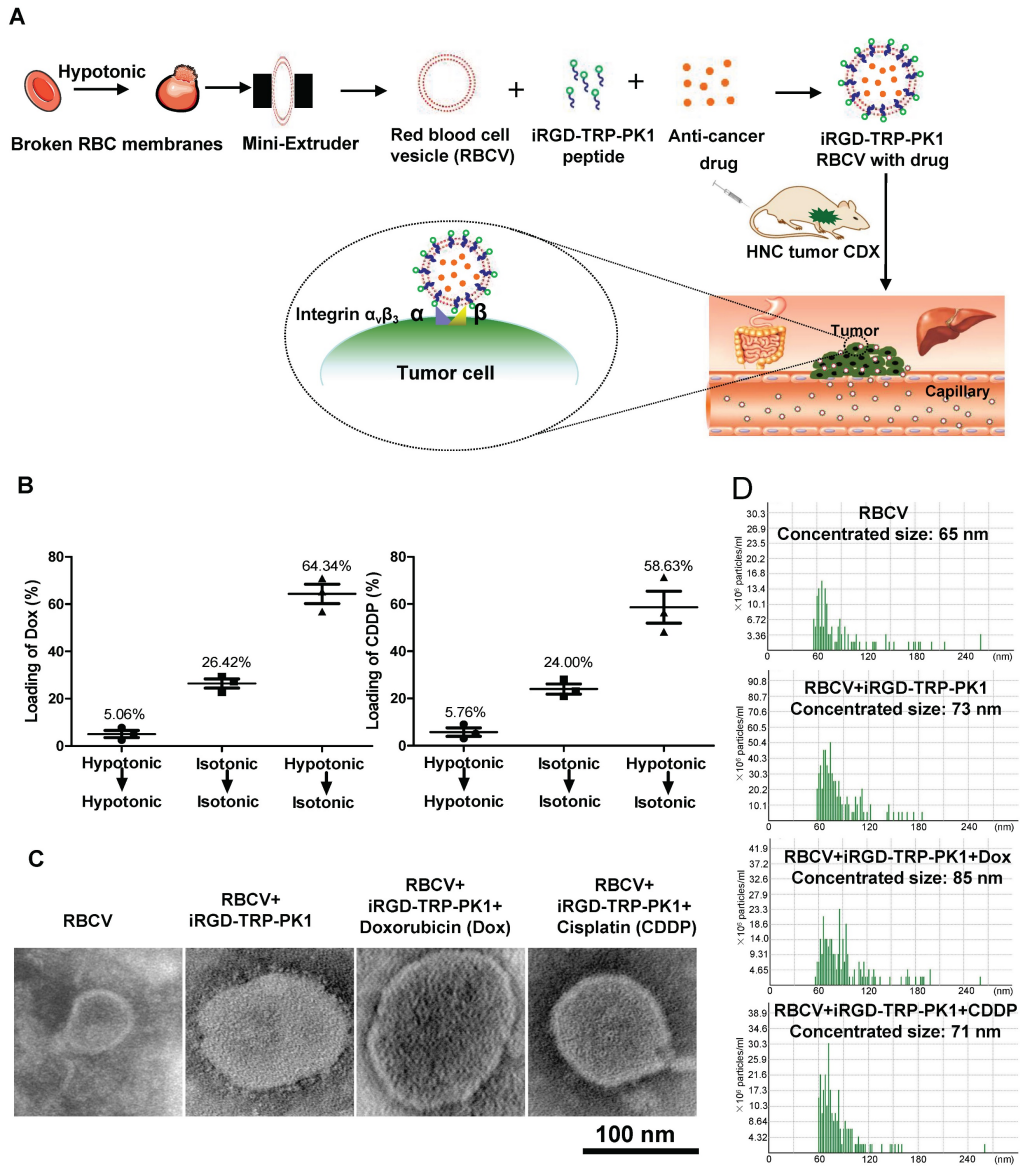
** Preparation and characterization of iRGD-TRP-PK1-modified RBCVs.** (A) Schematic illustration of the formation of iRGD-TRP-PK1-modified RBCVs. (B) Detection of Dox and CDDP loading efficiency by RBCVs in a hypotonic to isotonic transition environment. (C) Electron microscopy of iRGD-TRP-PK1-modified RBCVs and their loading with Dox and CDDP. (D) Particle size and concentration distribution of iRGD-TRP-PK1-modified RBCVs and their loading with Dox and CDDP. CDDP: cisplatin, Dox: doxorubicin, iRGD: internalizing RGD peptide, RBCV: red blood cell versicle.

To provide a comprehensive overview of our research, we created a process map outlining the study. Initially, RBCs were prepared and characterised as RBCVs. The results indicated that the RBCVs predominantly had a size of approximately 65 nm, a Zeta potential of -28.38 mV, and protein bands similar to those of intact RBCs **(Figure S1)**. iRGD-TRP-PK1 was then co-incubated with the RBCVs to facilitate attachment, followed by loading with chemotherapeutic agents. The resulting system was administered to mice, achieving targeted delivery of the chemotherapeutic drugs to the tumour site **(Figure 1A, sequence presented in Figure S2)**. To evaluate the physicochemical properties of the RBCV-targeted delivery system, we first assessed the loading efficiency of the chemotherapeutic drugs. The results showed that the loading efficiencies for Dox and CDDP were 64.34% and 58.63%, respectively, when using solutions with low to isotonic osmolarity. The optimal mixing ratio of Dox to RBCVs was determined to be 1 µM : 10^8^ particles **(Figure 1B, Figure S3)**. Morphological characterisation of RBCVs, iRGD-TRP-PK1-modified RBCVs and iRGD-TRP-PK1-modified RBCVs loaded with Dox or CDDP revealed that iRGD-TRP-PK1-modified RBCVs exhibited a "ring" structure on the surface, with black "dots" observed inside the RBCVs after drug loading **(Figure 1C)**. Size analysis indicated that iRGD-TRP-PK1-modified RBCVs were larger than free RBCVs, and further size increase was observed upon drug loading **(Figure 1D)**. These findings suggest that iRGD-TRP-PK1 binds effectively to the surface of RBCVs which are capable of loading chemotherapeutic agents.

### iRGD-TRP-PK1-modified RBCV target HNC cells

**Figure 2:**
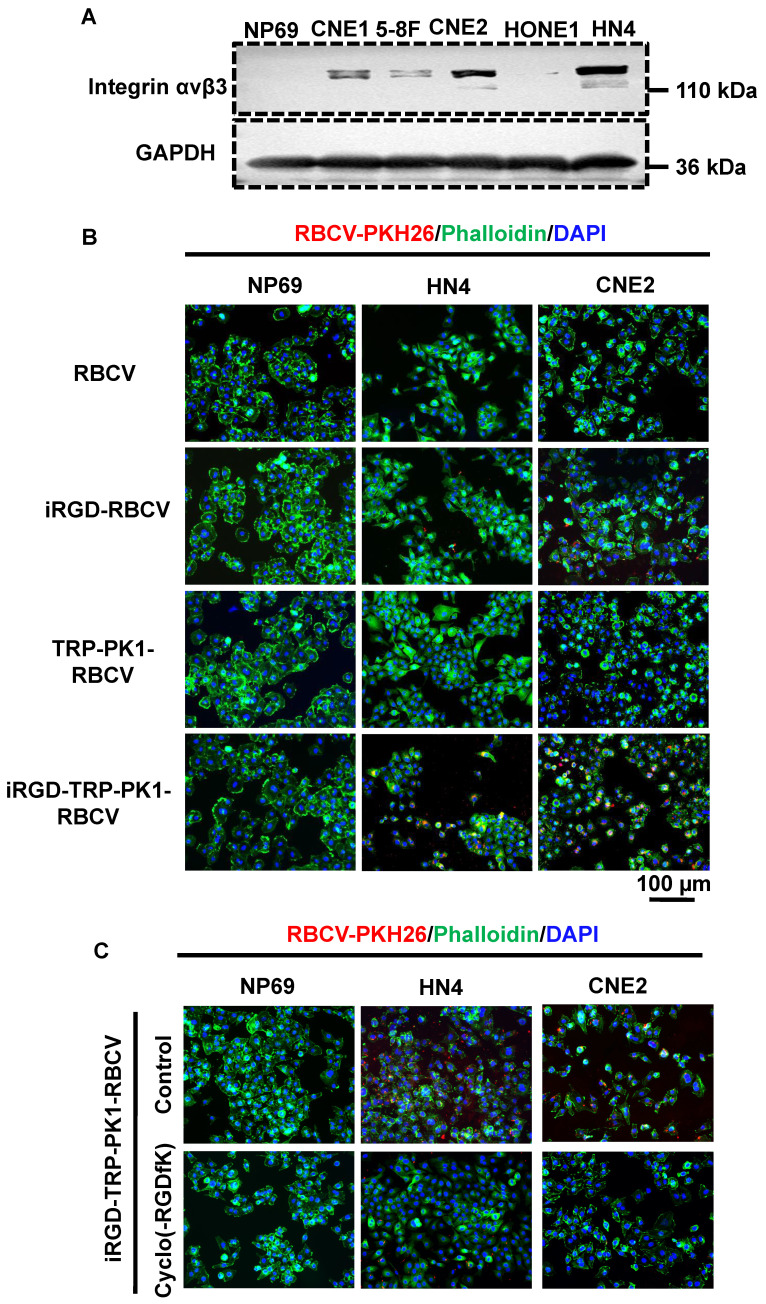

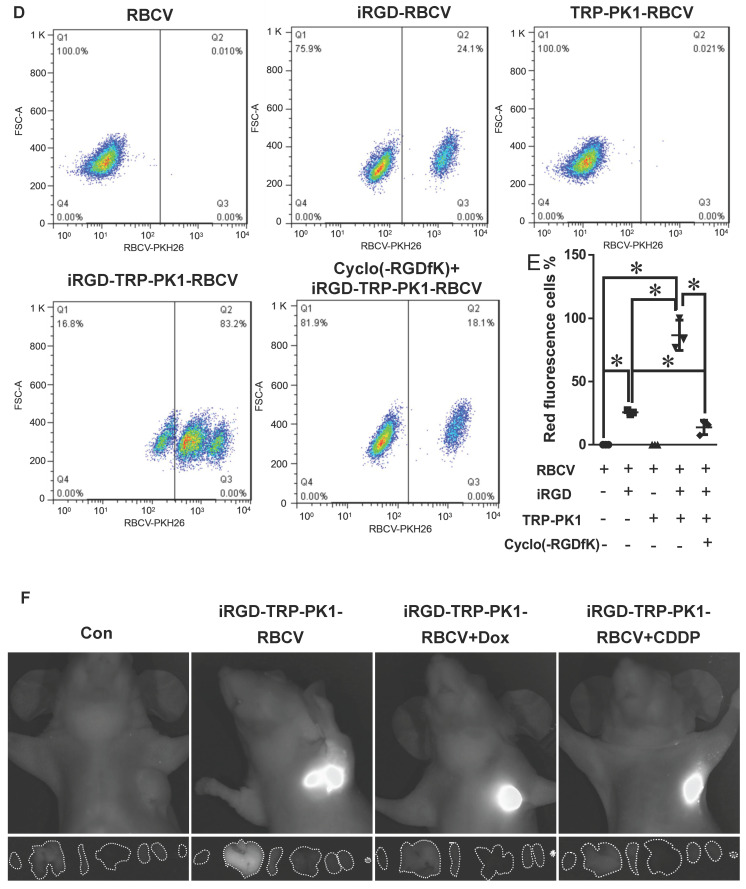
** iRGD-TRP-PK1 enhances the cellular uptake of RBCVs**. (A) Representative images of integrin α_v_β_3_ expression in NP69, CNE1, HN4, CNE2, HONE1, and 5-8F cells. (B) Representative images of iRGD-TRP-PK1-modified RBCVs uptake by NP69, HN4, and CNE2 cells. (C) Targeting of iRGD-TRP-PK1-modified RBCVs after integrin α_v_β_3_ inhibitor--Cyclo(-RGDfK) treatment. (D) Representative flow images of the fraction of RBCVs taken up by HN4 cells after treatment with iRGD-, TRP-PK1-, iRGD-TRP-PK1-modified RBCVs and Cyclo(-RGDfK) treatment. (E) Percentage of HN4 cells after uptake of RCBVs. (F) Targeting of iRGD-TRP-PK1-modified RBCVs and their loading with Dox and CDDP in tumors established by HN4 cells in a CDX model. CDX: cell-derived xenograft (CDX), iRGD: internalizing RGD peptide.

To assess the targeting ability of iRGD-TRP-PK1-modified RBCVs towards HNC cells, we first evaluated the expression of the iRGD peptide receptor α_v_β_3_ in HNC cells. HN4 and CNE2 cells, which exhibit high α_v_β_3_ expression, were selected for further studies **(Figure 2A)**. Cell experiments demonstrated significant uptake of iRGD or iRGD-TRP-PK1-modified RBCVs by HN4 and CNE2 cells **(Figure 2B)**. This uptake was effectively blocked by the integrin α_v_β_3_ inhibitor Cyclo(-RGDfK) **(Figure 2C)**.

PKH26-labelled RBCVs showed enhanced uptake by HN4 cells following modification with iRGD or iRGD-TRP-PK1, with the uptake percentage increasing from 25.7% to 86.63% for iRGD-TRP-PK1-modified RBCVs compared to iRGD-modified RBCVs. Cyclo(-RGDfK) treatment significantly reduced the uptake of iRGD- or iRGD-TRP-PK1-modified RBCVs, with the proportion of HN4 cells labelled dropping to approximately 13.76% **(Figure 2D-E)**.

To determine whether iRGD-TRP-PK1-modified RBCVs and their drug-loaded variants can target and accumulate in tumor tissues, we performed DiR labelling of RBCVs followed by tail vein injection into a cell-derived xenograft model. The results showed a significant increase in tumor tissues accumulation for iRGD-TRP-PK1-modified RBCVs, iRGD-TRP-PK1-modified RBCVs loaded with Dox, and iRGD-TRP-PK1-modified RBCVs loaded with CDDP **(Figure 2F)**. Additionally, Cyclo (-RGDfK) treatment reduced the targeting ability of iRGD-or iRGD-TRP-PK1-modified RBCVs for tumor tissues **(Figure S4)**. These findings strongly indicate that iRGD-TRP-PK1 significantly enhances the tumor-targeting capability of RBCVs, thereby facilitating the effective delivery of chemotherapeutic agents with targeting achieved through iRGD binding to α_v_β_3_ integrins.

### Self-release of anti-cancer drugs from iRGD-TRP-PK1-modified RBCV in HNC cells

**Figure 3:**
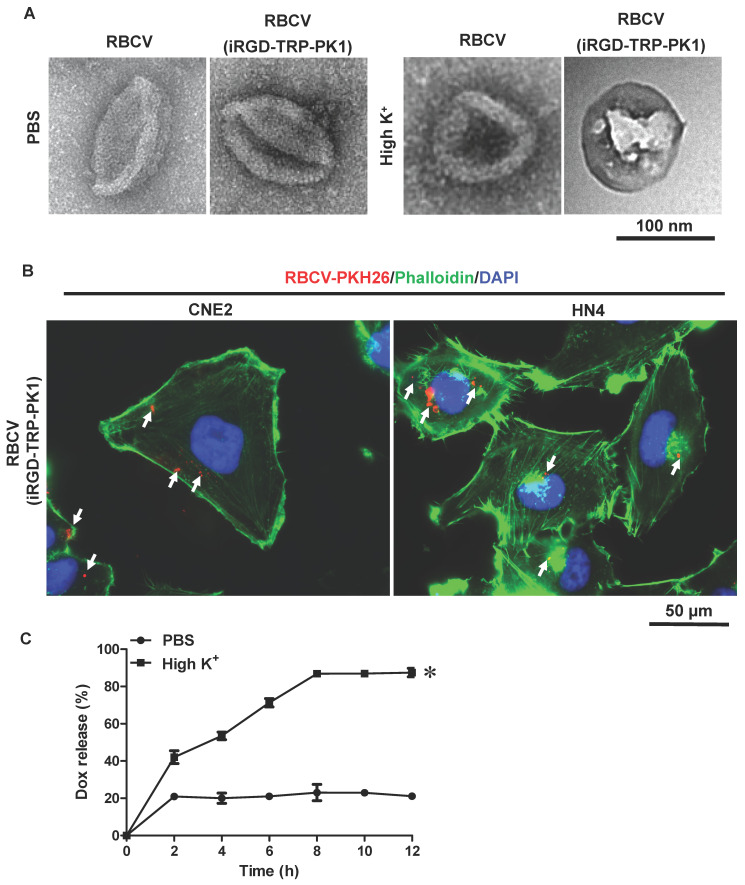

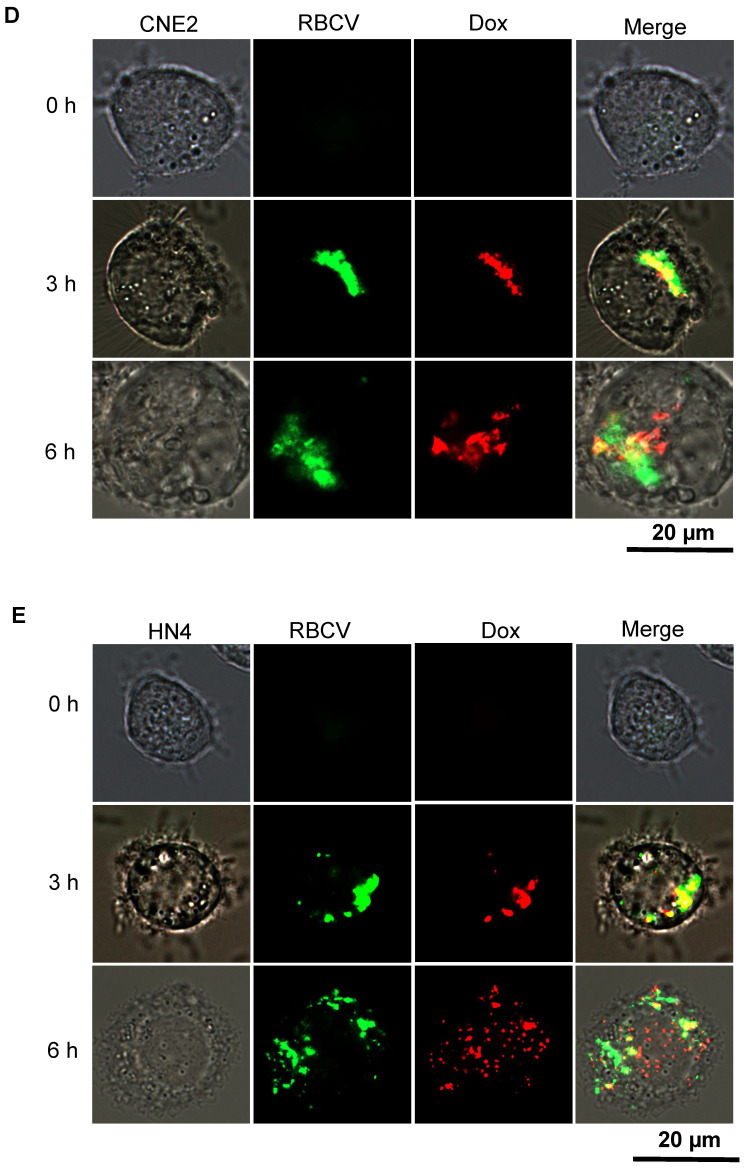
** iRGD-TRP-PK1 promoted anti-cancer drug release which loaded within RBCVs in HNC cells**. (A) Electron microscopy morphology images of iRGD-TRP-PK1-modified RBCVs in a high-potassium environment. (B) Representative images of iRGD-TRP-PK1-modified RBCVs distribution within HN4 and CNE2 cells. (C) Rate of Dox release in high K^+^ and PBS as time progresses, *P<0.05 by two-way ANOVA. (D) Release of Dox from iRGD-TRP-PK1-modified RBCVs loaded with Dox after co-incubation with CNE2 cells. (E) Release of Dox from iRGD-TRP-PK1-modified RBCVs loaded with Dox after co-incubation with HN4 cells. Dox: doxorubicin, iRGD: internalizing RGD peptide, RBCVs: red blood cell vesicles.

In addition to its capability to self-assemble onto lipid bilayers, TRP-PK1 also forms "leaky potassium" channels in biological membranes 15. Consequently, iRGD-TRP-PK1-modified RBCVs are expected to fragment due to this "leaky potassium" effect upon entering tumor cells, leading to the autonomous release of the loaded chemotherapeutic drugs. To verify whether iRGD-TRP-PK1-modified RBCVs can fragment in a high-potassium environment, we conducted further experiments. We first examined the morphology of RBCVs in a high-K^+^ buffer and observed significant fragmentation of iRGD-TRP-PK1-modified RBCVs under these conditions (**Figure 3A**). Additionally, 2D and 3D fluorescence microscopy revealed that iRGD-TRP-PK1-modified RBCVs were able to enter HN4 and CNE2 cells (**Figure 3B, Figure S5**). To further investigate the autonomous release of chemotherapeutic drugs, we used Dox, which exhibits autofluorescence, as a model. We analysed the release of Dox from RBCVs modified with iRGD-TRP-PK1 in high K^+^ conditions. The results showed that Dox was released more rapidly and in greater quantities from iRGD-TRP-PK1-modified RBCVs after high K^+^ treatment, and the release of Dox was not enhanced in high Na^+^ and high Ca^2+^ buffers **(Figure 3C, Figure S6)**. To confirm the intracellular release, we treated CNE2 and HN4 cells with iRGD-TRP-PK1-modified RBCVs loaded with Dox and observed the intracellular distribution of Dox (red) and RBCVs (green). We found that Dox co-localised with RBCVs inside the cells at 3 hours and began to separate around 6 hours (**Figure 3D-3E**). These observations suggest that iRGD-TRP-PK1-modified RBCVs can autonomously release their chemotherapeutic agents after entering into cells.

### iRGD-TRP-PK1-modified RBCV-loaded with chemotherapeutic agents significantly inhibits HNC cell proliferation and promotes apoptosis

**Figure 4:**
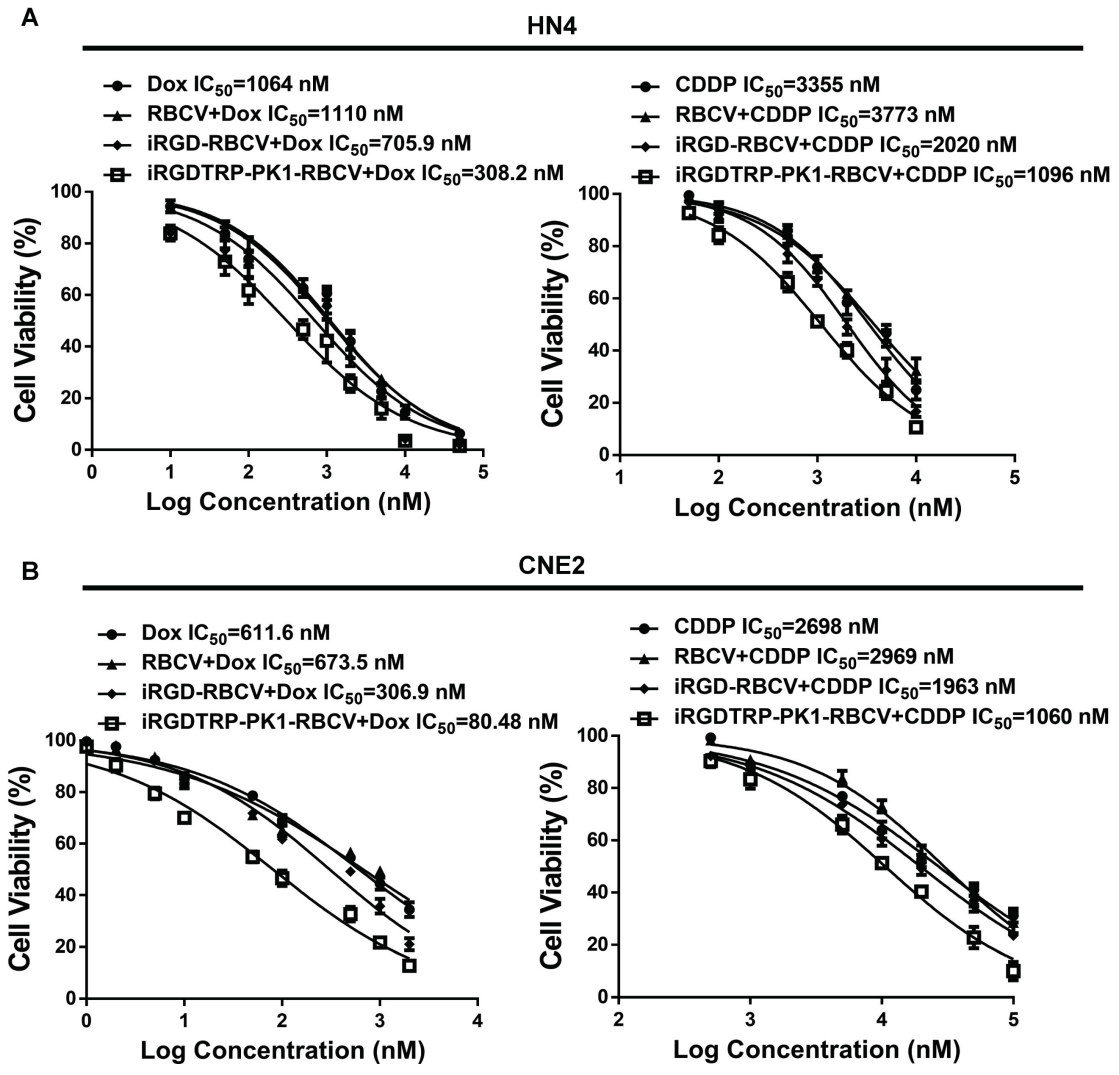

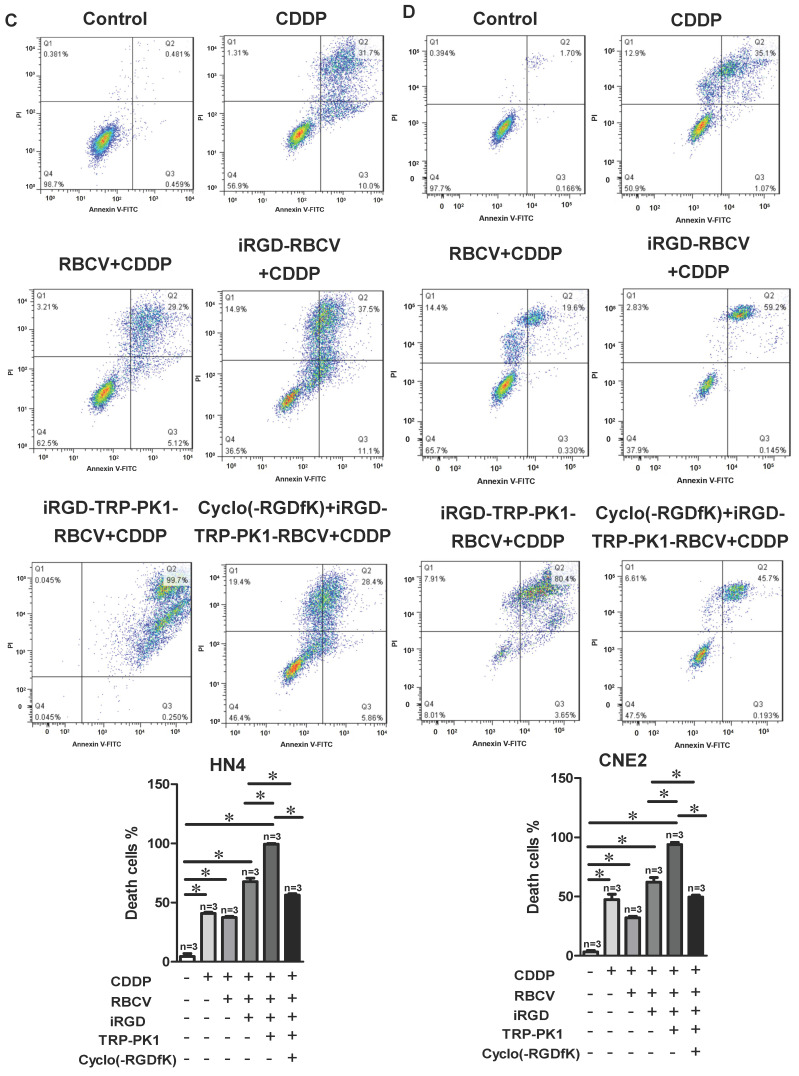
iRGD-TRP-PK1-modified RBCVs loaded with chemotherapeutic agents significantly inhibit HNC cell proliferation and promote apoptosis. (A) Inhibition efficiency assay of cells after treatment of HN4 cells with different concentration of Dox, RBCV+Dox, iRGD-RBCV+Dox, iRGD-TRP-PK1-RBCV+Dox or CDDP, RBCV+CDDP, iRGD-RBCV+CDDP, iRGD-TRP-PK1-RBCV+CDDP. (B) Inhibition efficiency assay of cells after treatment of CNE2 cells with different concentration of Dox, RBCV+Dox, iRGD-RBCV+Dox, iRGD-TRP-PK1-RBCV+Dox or CDDP, RBCV+CDDP, iRGD-RBCV+CDDP, iRGD-TRP-PK1-RBCV+CDDP. (C) Cell apoptosis of HN4 cells after treated with CDDP, RBCV+CDDP, iRGD-RBCV+CDDP, iRGD-TRP-PK1-RBCV+CDDP and Cyclo(-RGDfK)+iRGD-TRP-PK1-RBCV+CDDP. (D) Cell apoptosis of CNE2 cells after treated with CDDP, RBCV+CDDP, iRGD-RBCV+CDDP, iRGD-TRP-PK1-RBCV+CDDP and Cyclo(-RGDfK)+iRGD-TRP-PK1-RBCV+CDDP. CDDP: cisplatin, CCK-8: cell counting kit-8, Dox: doxorubicin, OD: optical density, iRGD: internalizing RGD peptide, RBCVs: red blood cell vesicles. *P < 0.05 by Student's t-test.

To evaluate the therapeutic efficacy of iRGD-TRP-PK1-modified RBCVs loaded with chemotherapeutic agents, we treated HN4 and CNE2 cells with iRGD-TRP-PK1-RBCVs loaded with Dox or CDDP. The results demonstrated that both iRGD-TRP-PK1-RBCVs+Dox and iRGD-TRP-PK1-RBCVs+CDDP significantly inhibited the proliferation of HN4 and CNE2 cells (**Figure S7A-S7B**). Notably, iRGD-TRP-PK1-modified RBCVs loaded with Dox exhibited superior inhibition of cell proliferation compared to free Dox, RBCVs loaded with Dox, or iRGD-modified RBCVs loaded with Dox **(Figure 4A-4B)**. Apoptosis assays revealed that iRGD-TRP-PK1-RBCVs loaded with Dox or CDDP significantly enhanced apoptosis in HN4 and CNE2 cells (**Figure S7C-S7F**). Flow cytometry further demonstrated that iRGD-TRP-PK1-modified RBCVs loaded with CDDP had a stronger ability to induce apoptosis in HN4 and CNE2 cells compared to free CDDP, RBCV-loaded CDDP, and iRGD-modified RBCV-loaded CDDP. This enhanced apoptosis was inhibited by Cyclo(-RGDfK) treatment **(Figure 4C-4D)**. These findings indicate that iRGD-TRP-PK1-modified RBCVs loaded with chemotherapeutic drugs exert significant anti-tumor effects through targeting α_v_β_3_.

### iRGD-TRP-PK1-modified RBCV increases tumor suppression and reduces the side effects of chemotherapy drugs

**Figure 5:**
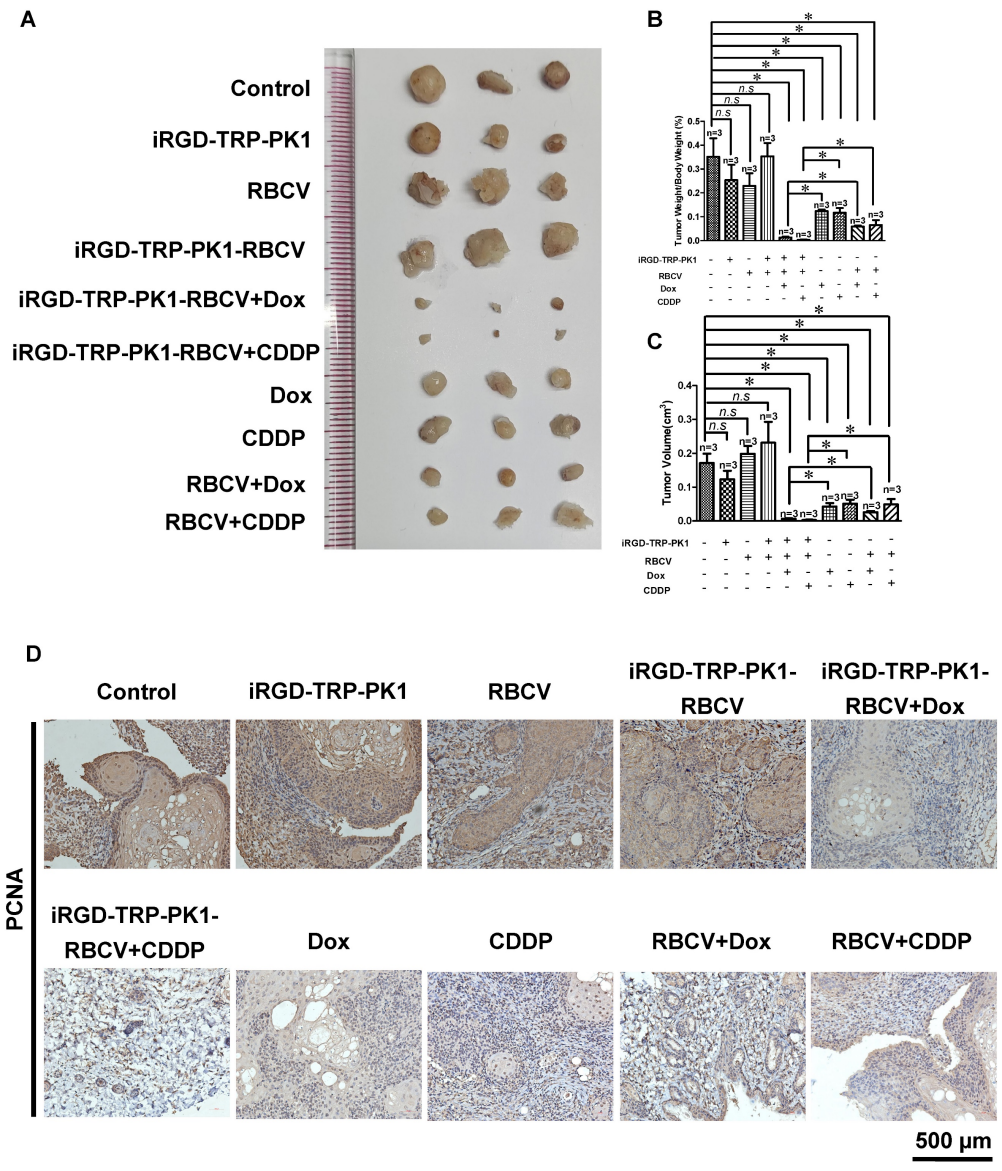

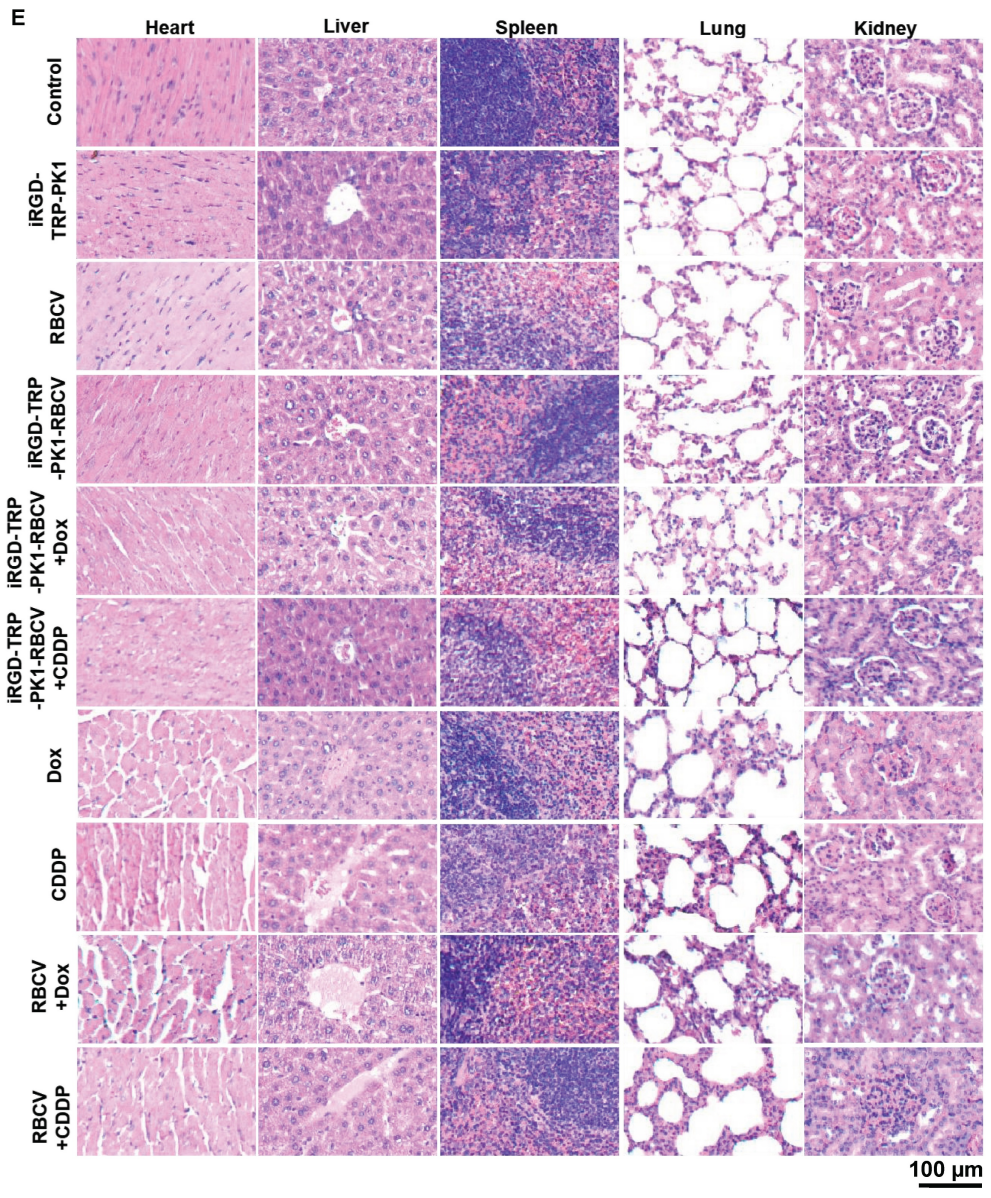
iRGD-TRP-PK1-modified RBCVs loaded with chemotherapeutic agents significantly increase tumor suppression and reduce side effects. (A) Representative images depicting tumors after treatment with different formulations. (B) Statistical analysis of tumor weight/body weight after treatment with different formulations. (C) Statistical analysis of tumor volume after treatment with different formulations. (D) Representative images of PCNA expression by IHC. (E) Toxic analysis of organs from mice after treatment with different formulations by the pathological HE staining. HE: hematoxylin and eosin, iRGD: internalizing RGD peptide, RBCVs: red blood cell vesicles. *P < 0.05 using Student's t-test.

To evaluate the efficacy of iRGD-TRP-PK1-modified RBCVs in targeting tumors and mitigating the side effects of chemotherapeutic drugs, we administered Dox or CDDP either as free drugs or encapsulated within iRGD-TRP-PK1-RBCVs via tail vein injection into HN4-derived xenograft models. The results demonstrated that iRGD-TRP-PK1-RBCVs loaded with Dox or CDDP significantly inhibited tumour growth compared to free Dox, free CDDP, RBCVs+Dox, and RBCVs+CDDP treatments (**Figure 5A-5C**). IHC analysis of proliferating cell nuclear antigen (PCNA) revealed that treatment with iRGD-TRP-PK1-RBCVs loaded with Dox or CDDP effectively reduced PCNA expression in tumour tissues **(Figure 5D)**. Additionally, we assessed the *in vivo* systemic toxicity of iRGD-TRP-PK1-RBCVs loaded with chemotherapeutic agents through morphological analysis. Hematoxylin and eosin staining of major organs—including the heart, liver, spleen, lungs, and kidneys—showed no significant pathological changes in tumour-bearing mice treated with iRGD-TRP-PK1-RBCVs+Dox or iRGD-TRP-PK1-RBCVs+CDDP (**Figure 5E**). These findings suggest that iRGD-TRP-PK1-modified RBCVs, when loaded with chemotherapeutic drugs, effectively target tumour tissues, enhance tumour cell killing, and minimise drug accumulation in normal tissues, thereby reducing the associated side effects.

## Discussion

Chemotherapy remains one of the most commonly employed treatments in clinical practice. However, the severe toxic side effects associated with chemotherapeutic drugs significantly limit their widespread use. For instance, drugs like Dox and CDDP are frequently used in clinical settings but are known to cause severe cardiotoxicity, gastrointestinal reactions, neurotoxicity, and myelosuppression, with long-term exposure, posing significant challenges for patients undergoing chemotherapy 19-23. Addressing issues such as lack of specificity, chemical resistance, and severe side effects by merely increasing drug dosage is not a viable solution. In this context, nanotechnology presents promising avenues for improving drug delivery and enhancing sensitivity. In this study, we developed a nano-targeted delivery system based on iRGD-TRP-PK1 and RBCVs. This system effectively targets integrin α_v_β_3_-overexpressing HNC cells and facilitates the controlled release of encapsulated chemotherapeutic drugs upon targeting these cells. Consequently, iRGD-TRP-PK1-RBCVs loaded with chemotherapeutic agents not only enhance tumour cell killing but also mitigate the adverse side effects associated with these drugs.

Intravenous injection is a primary method for delivering chemotherapeutic drugs wherein the drugs are administered through peripheral or central venous drip, circulate to the heart, and are gradually distributed throughout the body to achieve therapeutic concentrations 24. This approach necessitates high doses, often leading to significant side effects for patients 24. Targeting chemotherapy drugs directly to tumour sites could improve treatment efficacy while reducing systemic side effects. Nanoparticle systems, capable of carrying substantial drug loads, offer an excellent means of targeted drug delivery 25. RBCVs, derived from red blood cells, are renowned for their exceptional, biocompatibility, low immunogenicity, evasion of immune system clearance, and extended blood circulation. The lack of a nucleus and organelles in erythrocytes facilitates their extraction and purification 26-31. As such, erythrocyte membrane-based nanocarriers have attracted considerable attention in drug delivery applications 32,33. Loading drugs into RBCVs is typically achieved by manipulating osmolarity 34,35.

Nanoparticles, including RBCVs, can accumulate in various tissues, such as the heart, spleen, lungs, liver, and kidneys through systemic circulation 36,37. Therefore, enhancing the enrichment of RBCVs at tumor sites is crucial. iRGD is frequently utilised to decorate erythrocyte membranes for targeted delivery 38,39. iRGD exhibits an affinity for integrin-αvβ3, which is highly expressed in many tumor cells 40. Additionally, iRGD-modified nanoparticles including liposomes can activate endocytosis, thereby enhancing the tumour-killing effects of the encapsulated drugs 41,42. Common methods for iRGD modification of nanoparticles such as RBCVs include genetic engineering, post-extraction modifications, and membrane fusion. However, these methods often present challenges such as complexity, high costs, susceptibility to toxic chemicals, or inefficacy 43-45. In this study, we utilised the S4 peptide from the cell's self-expressed TRPV4 protein, known as TRP-PK1, which was conjugated to iRGD. This peptide was then used to incorporate iRGD into the RBCV lipid bilayer. The iRGD is designed to integrate into the RBCV following a brief co-incubation. Notably, the size of the RBCVs increased after incubation, and their volume further expanded upon loading with chemotherapeutic drugs. This volumetric increase aligns with findings from other vesicle studies, such as those involving exosomes 12. This design is efficient, easy to implement, and well-suited for clinical applications.

The drug is contained within the particles 46-48. Therefore, in addition to their targeting properties, nanosystems play a crucial role in drug release as well as targeting. For instance, Ge *et al*. employed electric field-responsive hydrogels to release drugs at specific sites through the application of electric fields 49. Similarly, Wu *et al*. developed an albumin-derived core-shell Dox drug delivery system that facilitates drug release triggered by pH changes and proteolytic enzymes 50. However, these methods involve additional procedures or the introduction of extra substances, which may lead to unintended side effects. In our research, we relied on the "leaky potassium" channel activity of TRP-PK1 for drug release. This channel selectively transports K^+^ from areas of high to low concentration. iRGD-TRP-PK1-modified RBCVs are internalized by tumor cells, where the high intracellular K^+^ concentration causes the RBCVs to rupture releasing the encapsulated chemotherapeutic drugs. This approach leverages TRP-PK1's unique property as a "leaky potassium" channel to introduce an innovative drug release mechanism.

The ultimate objective of designing targeted nanoparticles is to effectively kill tumors while minimizing drug toxicity 45,51. Our evaluation of HNC cell growth both *in vitro* and *in vivo*, after loading chemotherapeutic agents onto iRGD-TRP-PK1-RBCVs revealed that the combination of iRGD, TRP-PK1, RBCVs, and chemotherapeutic drugs resulted in a significantly enhanced tumor-killing effect and reduced drug toxicity.

## Conclusion

This study reveals that TRP-PK1 peptide aids in the autonomous insertion of iRGD into RBCVs, enhancing their targeting ability. Additionally, TRP-PK1 self-assembles into a "potassium leaky" channel upon uptake by tumor cells, promoting the fragmentation of RBCVs and the subsequent release of encapsulated chemotherapeutic drugs. Consequently, the targeted release of chemotherapeutic drugs to kill tumor cells was achieved. This research not only introduces novel strategies for HNC treatment but also offers insights and potential methodologies for targeted delivery and release in other diseases.

## Supplementary Material

Supplementary figures.
