# Supplementary material for: iRGD-TRP-PK1-modified red blood cell membrane vesicles as a new chemotherapeutic drug delivery and targeting system in head and neck cancer
Source: Theranostics. 2025 Jan 1;15(1):86–102. doi: 10.7150/thno.99481 (PMC11667238; doi:10.7150/thno.99481)
Supplement: Supplementary file 1 — Supplementary figures. [file thnov15p0086s1.pdf]

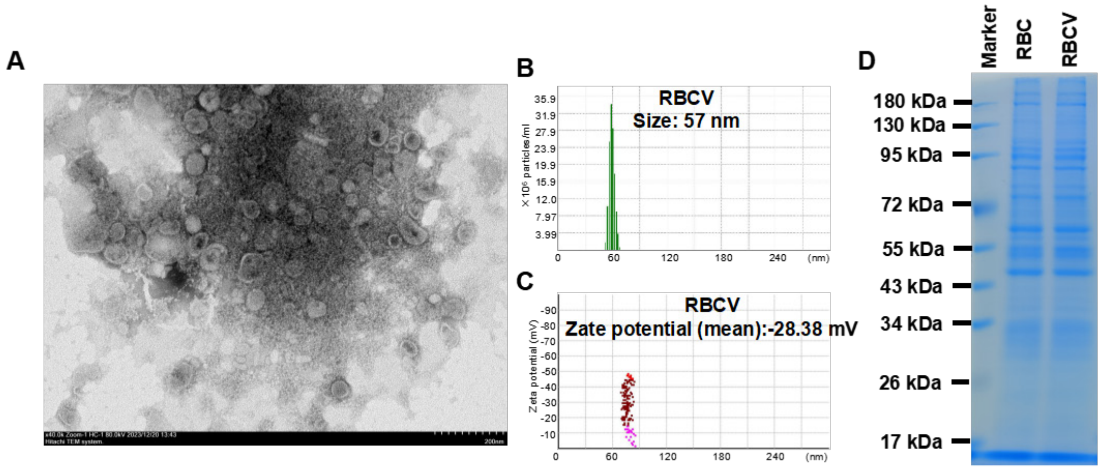

Figure S1. Identification of red blood cell vesicles. (A) Representative picture of RBCV TEM analysis. (B) RBCV particle size analysis. (C) Zeta potential analysis of RBCV. (D) Polyacrylamide gel electrophoresis of RBC and RBCV.

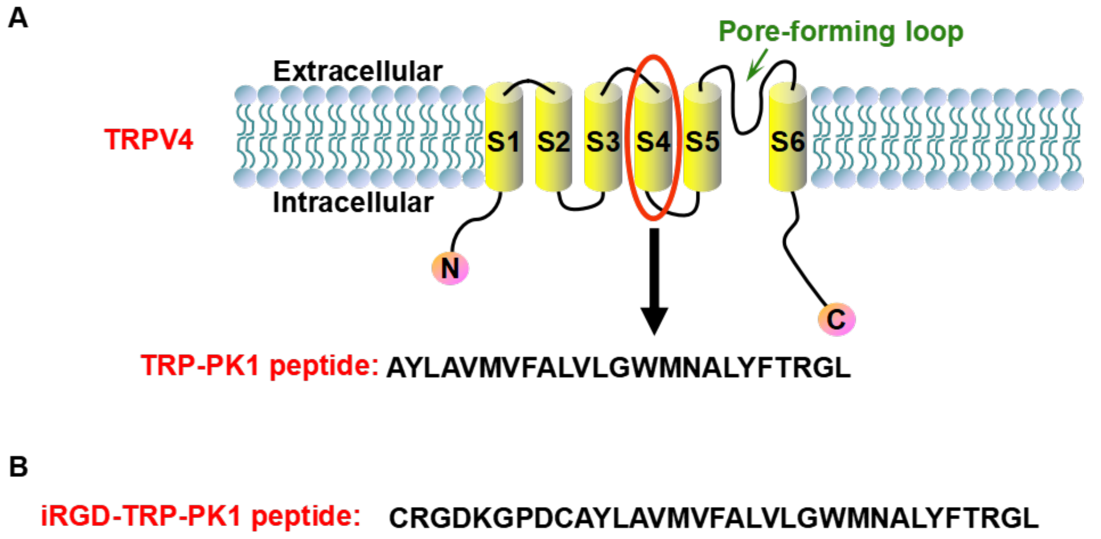

Figure S2. iRGD-TRP-PK1 and TRP-PK1 peptide sequence. (A) TRP-PK1 peptide location in TRPV4 and sequence. (B) Sequence of iRGD-TRP-PK1 peptide construction. iRGD: internalizing RGD peptide, TRPV4: transient receptor potential vanilloid 4.

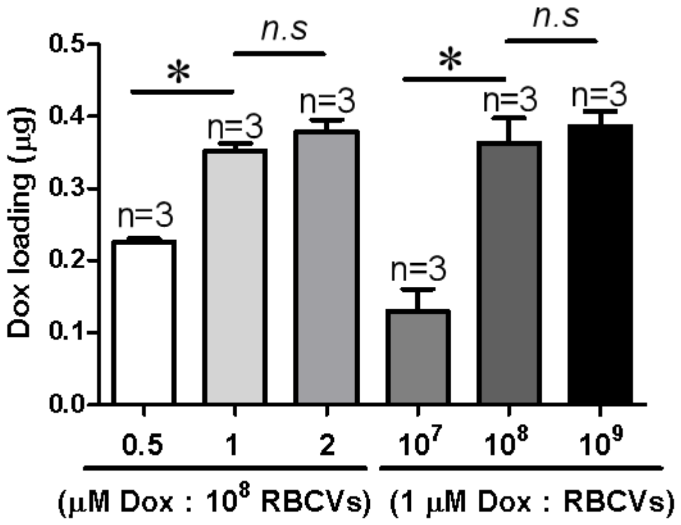

Figure S3. The amount of Dox being loaded after mixing different proportions of Dox with RBCVs.

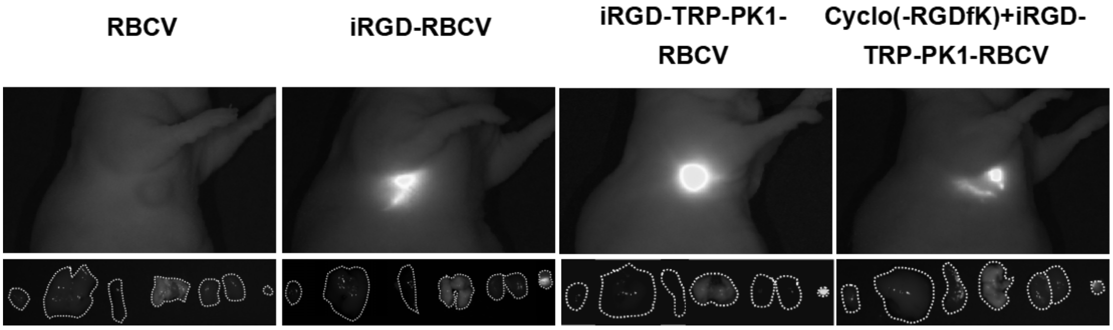

Figure S4. Targeting of iRGD-, iRGD-TRP-PK1-modified RBCVs and iRGD-TRP-PK1-modified RBCVs after Cyclo(-RGDfK) treated in tumors established by HN4 cells in a CDX model.

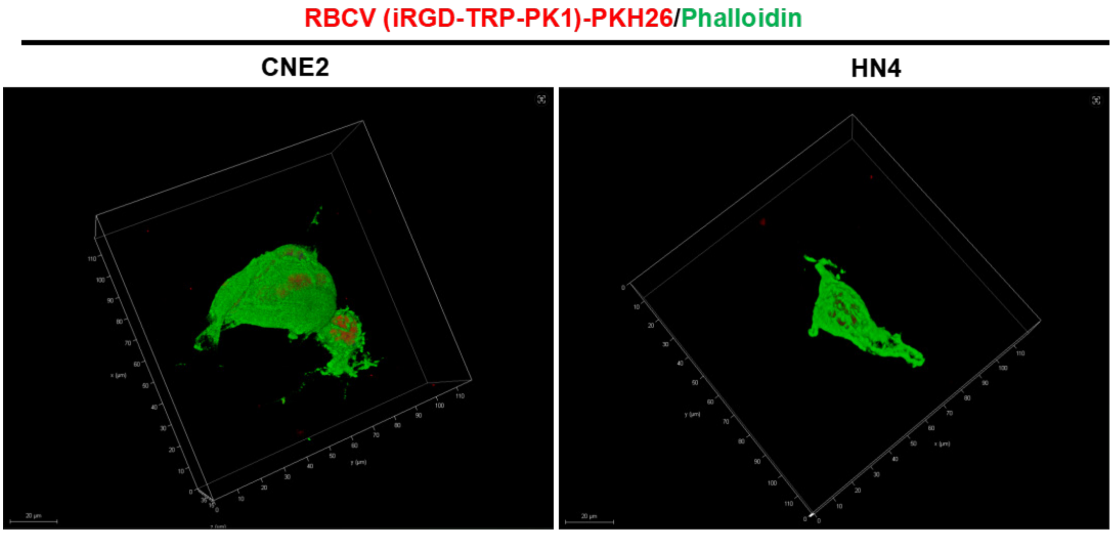

Figure S5. Representative 3D images of iRGD-TRP-PK1-modified RBCVs distribution within HN4 and CNE2 cells.

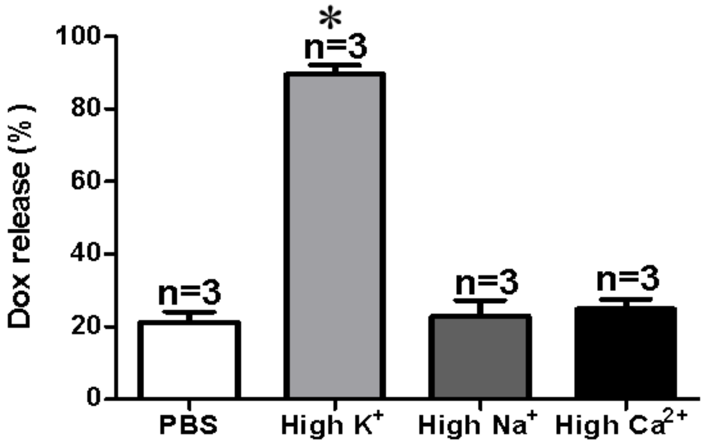

Figure S6. iRGD-TRP-PK1-modified RBCV loaded with Dox and the rate of Dox release after 12 h of treatment using PBS, high K^+ (60 mM), high Na^+ (140 mM), and high Ca^{2+} (5 mM) buffers.

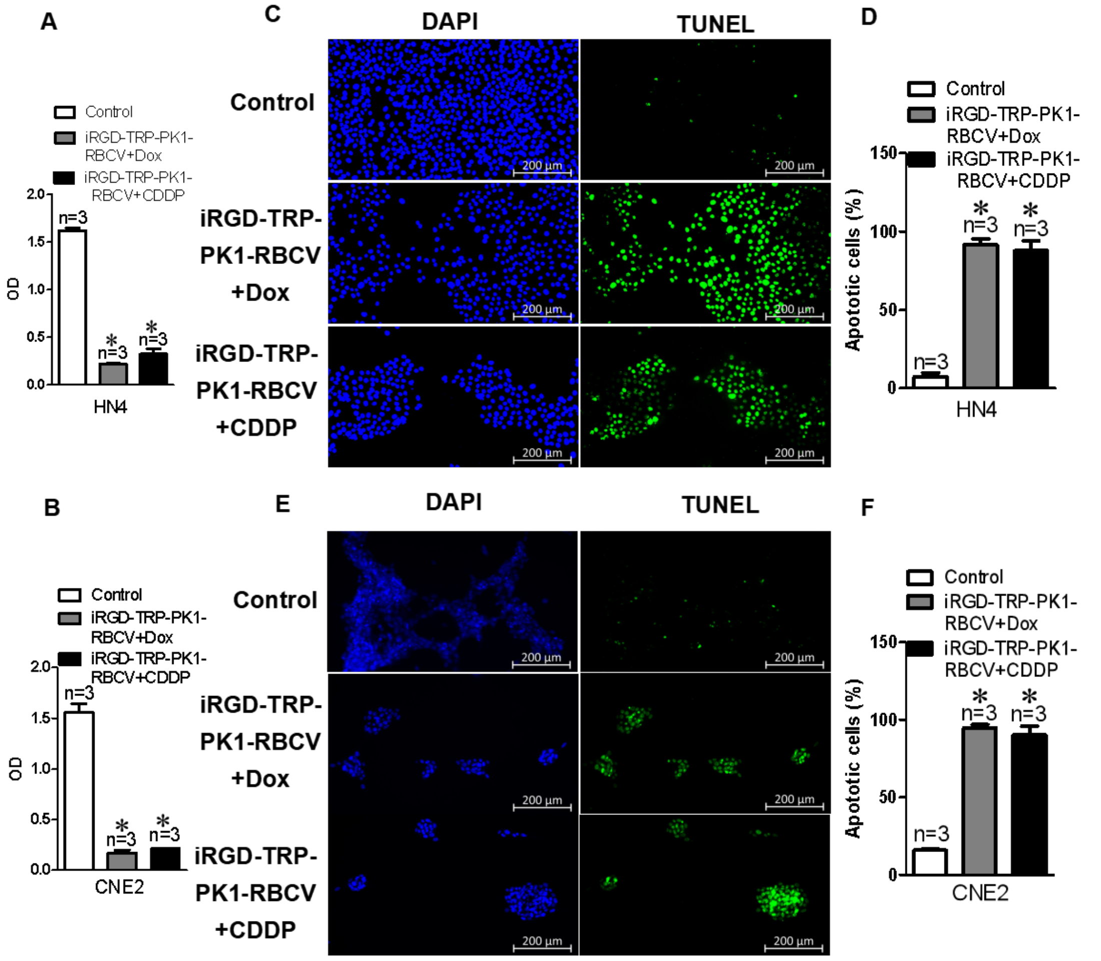

Figure S7. iRGD-TRP-PK1-modified RBCVs loaded with chemotherapeutic agents significantly inhibit HNC cell proliferation and promote apoptosis. (A) CCK-8 quantification assay results after treatment with iRGD-TRP-PK1-modified RBCVs loaded with Dox and CDDP in HN4 cells. (B) CCK-8 quantification assay results after treatment with iRGD-TRP-PK1-modified RBCVs loaded with Dox and CDDP in CNE2 cells. (C) Representative images of cell apoptosis after treatment with iRGD-TRP-PK1-modified RBCVs loaded with Dox and CDDP in HN4 cells. (D) Statistical analysis of HN4 cells apoptosis after treatment with iRGD-TRP-PK1-modified RBCVs loaded with Dox and CDDP. (E) Representative images of cell apoptosis after treatment with iRGD-TRP-PK1-modified RBCVs loaded with Dox and CDDP in CNE2 cells. (F) Statistical analysis of CNE2 cells apoptosis after treatment with iRGD-TRP-PK1-modified RBCVs loaded with Dox and CDDP. CDDP: cisplatin, CCK-8: cell counting kit-8, Dox: doxorubicin, HNC: head and neck cancer, OD: optical density, iRGD: internalizing RGD peptide, RBCVs: red blood cell vesicles. *$P < 0.05$ by Student's t-test.
